# Temporal Dynamics of Subtle Cognitive Change: Validation of a User-Friendly Multidomain Digital Assessment Using an Alcohol Challenge

**DOI:** 10.2196/55469

**Published:** 2025-06-12

**Authors:** John Frederick Dyer, Florentine Marie Barbey, Ayan Ghoshal, Ann Marie Hake, Bryan J Hansen, Md Nurul Islam, Judith Jaeger, Rouba Kozak, Hugh Marston, Mark Moss, Viet Nguyen, Rebecca Louise Quinn, Leslie A Shinobu, Elizabeth Tunbridge, Brian Murphy, Niamh Kennedy

**Affiliations:** 1 Cumulus Neuroscience Belfast United Kingdom; 2 Cumulus Neuroscience Dublin Ireland; 3 Biogen Inc Cambridge, MA United States; 4 Eli Lilly and Company Cambridge, MA United States; 5 Janssen Research & Development, LLC, a Johnson & Johnson company Philadelphia, PA United States; 6 CognitionMetrics Wilmington, DE United States; 7 Neuroscience Translational Medicine Takeda Pharmaceuticals Cambridge, MA United States; 8 CNS Diseases Research Boehringer Ingelheim Biberach Germany; 9 Department of Anatomy and Neurobiology Chobanian & Avedisian School of Medicine Boston University Boston, MA United States; 10 University of Ulster Coleraine United Kingdom; 11 Neuro TRC Bristol-Myers Squibb Cambridge, MA United States

**Keywords:** cognitive testing, mobile device, decentralized trials, alcohol challenge, sensitivity to change

## Abstract

**Background:**

Clinical trials in neurological and psychiatric indications are hampered by poor measurement fidelity in currently used “standardized” rating scales. Digital, repeatable tests that can be remotely administered offer a more fine-grained understanding of the patient’s clinical trajectory. Several such tools are being developed, but only a few have been validated in terms of their ability to discern and describe change over time—a critical element of clinical trials.

**Objective:**

Four cognitive tasks from a digital battery (delivered via tablet) are administered at high frequency following an alcohol challenge to assess sensitivity to change. The tasks are novel, repeatable, and self-administered implementations of classic neurobehavioral paradigms.

**Methods:**

Thirty healthy younger adults were assessed on 2 separate days, once under the influence of alcohol and once under a placebo, with order counterbalanced. Each day included 8 assessments. The tasks comprised novel, engaging implementations of the Digit Symbol Substitution Task (DSST), reaction time, N-back working memory, and visual associative/episodic memory, and were compared with benchmark measures. In-laboratory assessments were preceded by massed practice (3 sessions), and blood alcohol concentration was monitored throughout using a breathalyzer and a Visual Analog Scale.

**Results:**

Alcohol-related impairment was observed across multiple measures, followed by a return to baseline as blood alcohol concentration declined. A slight practice effect was noted between the first and second sessions for the digital DSST, along with a longer-term effect across the 2 days. Moderate to strong correlations between digital and benchmark measures were observed at peak intoxication.

**Conclusions:**

Under alcohol challenge, this battery, along with benchmark standardized tests, demonstrates sensitivity to subtle changes in cognitive performance over time. Practice effects are minimal within this condensed protocol. Patient-friendly, repeatable tests administered via a digital platform, such as those in the current battery, warrant further investigation in the context of remote clinical studies that require methodological approaches capable of discerning and describing small changes over time. The availability of validated single tests or test batteries as sensitive tools that can be easily and frequently administered (eg, daily) would address a critical gap: the lack of descriptors with sufficient sensitivity, specificity, and reliability to detect cognitive changes over time in clinical trials of new therapies for neurological and psychiatric conditions.

## Introduction

Cognitive function is pivotal to quality of life and is compromised across a wide range of disorders. This includes conditions with primary pathology anchored in the central nervous system (CNS—ie, neurodevelopmental, neuropsychiatric, and neurodegenerative disorders, as well as trauma), as well as CNS impairment secondary to other drivers of brain dysfunction (eg, “brain fog” following chemotherapy, chimeric antigen receptor T-cell, or radiation therapy, COVID-19).

Well-established and accepted tools in the neurodegenerative domain (eg, Mini-Mental Status Examination [MMSE], Montreal Cognitive Assessment [MoCA], the Alzheimer’s Disease Assessment Scale—Cognitive Subscale [ADAS-Cog], and Clinical Dementia Rating [CDR] in Alzheimer disease [AD]) exist, but are—to varying extents—burdensome, error-prone, and, perhaps most importantly, relatively insensitive to small yet clinically significant changes in cognitive function, especially in early disease [1]. To highlight the clinically meaningful impact of changes in cognitive function between untreated and treated disease states, tools are needed that can detect changes that may appear subtle on current instruments. These new tools must also be suitable for the growing scale and complexity of clinical trials [2,3]. In the context of dementia, a clinically meaningful difference has been benchmarked by Borland [4] as a decrease of 3.8 points (rounded to 4 in clinical practice) on the Symbol Digit Modalities Test (SDMT). This corresponds to a change of at least 0.5 on the CDR (Sum of Boxes score) for a patient who was unimpaired at baseline. The relevance of digital cognitive batteries in accelerating clinical research in dementia and other neurodegenerative conditions is widely recognized [5]. Digitized end points—enhanced for repeatability (as in this study) and at-home longitudinal assessment—could reduce the burden on patients and clinicians (ie, patients would no longer need to travel for a potentially stressful face-to-face test in an unfamiliar environment), while providing more reliable insights into cognitive change [6].

Cognitive impairment is also an underappreciated feature of various psychiatric conditions, such as schizophrenia, major depressive disorder (MDD), and obsessive-compulsive disorder, and is one that patients often cite as having a significant impact on quality of life [7-9]. Cognition is now recognized in psychiatry as a primary treatment target [10,11]. However, there remains limited consensus on appropriate (digital) measurement tools in this domain (see literature review results in [11]). While successful demonstrations are beginning to emerge—for example, the paper-based Digit Symbol Substitution Task (DSST) was recently used as a primary end point to evaluate the procognitive effects of vortioxetine in MDD [12]—CNS clinical trial results have at times been complicated by strong placebo-like effects on cognition, possibly due to the impact of increased clinical scrutiny during trial participation [13].

An ideal cognitive measurement tool for CNS clinical trials should be sensitive to both the presence of and changes in the underlying neurological or disease state, while also accounting for day-to-day variations that introduce within-participant noise in standard drug versus placebo comparisons. This can be addressed through high-frequency “burst measurement,” which enables the estimation of a stable individual baseline by aggregating data across multiple temporally close time points [14-16]. Burst measurement is not possible or practical with traditional end point measures, which are typically conducted in highly controlled, artificial settings and lack repeatability—partly due to the limited availability of alternate forms and the influence of practice effects [17]. Moreover, the burden and cost of administering a full cognitive assessment would likely make such an approach unrealistic.

One possible solution to this issue is the development of a digital platform with automated administration/scoring, designed for remote data collection—eliminating the need for participants to visit a clinic [18]. By leveraging data aggregation and improved measurement reliability, so-called decentralized clinical trials may detect changes due to drug intervention (or a lack of decline, as the case may be) earlier than the current model, with fewer participants, or a combination of both. Stably measured change within individuals can serve as the outcome variable, rather than the relatively noisier group mean. Many digital measurement tools/platforms have been proposed (see [19,20] for several examples), but only a few have been explicitly validated for their ability to detect subtle changes in cognitive performance over time. For instance, some tests in the digital CogState battery have been assessed for sensitivity to alcohol- and fatigue-induced impairment [21], and separately, for practice effects in a high-frequency assessment study [22]. However, in those cases, the tests used were originally developed for administration by a trained experimenter/rater. Other such tools have been used to measure change over time at much longer intervals—for example, at 6-month intervals due to disease ([23]—Altoida) or drug effect ([24]—Cambridge Cognition).

The Cumulus Neuroscience cognitive assessment platform was developed in collaboration with a precompetitive consortium of pharma companies, including Biogen, Eli Lilly, Johnson & Johnson, Takeda, Boehringer Ingelheim, and Bristol Myers Squibb. Experts in CNS clinical development, digital end points, and cognitive testing worked together to design a suite of tasks for use in both remote/home- and clinic-based research, intended to be self-administered by nontechnical participants as well as those with cognitive impairments [16,25]. “Appification” and limited gamification approaches were used to encourage continued and genuine engagement with the tasks, helping to ensure the validity of repeated remote samples. The consortium also designed a series of studies, including this one, to validate the approach across a range of cohorts and settings.

The assessment platform can be used to administer evaluations across multiple modalities, including electroencephalography (EEG), cognition, mood, and language. It has previously been field tested for at-home feasibility in a high-frequency protocol with healthy older adults [25] and in the context of a ketamine challenge involving burst measurement alternating between the laboratory and home in younger healthy adults [16]. The platform has also been validated in an AD dementia cohort [26], and separately in patients with amyotrophic lateral sclerosis and frontotemporal dementia [27].

In this validation study, we explore the use of a battery of 4 tests expected to be informative for characterizing baseline performance and detecting change following intervention(s) in the context of neurodegeneration and psychiatry. The assessed domains include episodic memory, executive function, working memory, and psychomotor speed [28]. Specifically, the 4 tests are (1) a DSST; (2) a visual associative learning test; (3) a simple reaction time test; and (4) a visual N-back. We investigate the sensitivity of tasks to acute impairment and recovery. An alcohol challenge is used as an ethically acceptable method to induce well-characterized, clinically meaningful levels of cognitive dysfunction (target blood alcohol concentration [BAC%] 0.08-0.1). This BAC level slightly exceeds the UK drink-driving limit (0.08). BAC% levels within this range have been shown to produce somewhat selective and temporary deterioration in several cognitive domains [21,29,30], including episodic memory [31,32], learning [33,34], attention [35], executive function [36,37], and reaction time [38]. In healthy young adults, such changes are also unlikely to be captured by standard cognitive clinical end points (eg, MMSE, MoCA), due to ceiling effects.

This study uses high measurement frequency—up to 8 repetitions per day, depending on the task—to track the cognitive dynamics of alcohol intoxication. Alongside the Cumulus tasks, several well-validated benchmark tests are included for comparison, including the paper-based DSST (Wechsler Adult Intelligence Scale [WAIS] IV version), the Verbal Paired Associates—Part I (VPA-I), and the Cambridge Neuropsychological Test Automated Battery (CANTAB) Paired Associates Learning (PAL).

Our hypotheses are as follows: (1) statistically significant effects of alcohol administration will be observed on test performance; (2) practice effects will stabilize within an initial massed practice period (3 sessions); and (3) concurrent validity will be demonstrated between Cumulus digital tasks and their “benchmark” equivalents, as assessed via correlation analysis.

## Methods

### Ethical Considerations

This study was conducted following approval from the Ulster University School of Psychology Research Ethics Filter Committee, United Kingdom (REC code FCPSY-21-025-A). All participants provided informed consent to participate in the study after reading an information sheet and having the opportunity to ask questions about the procedure. Recruitment was carried out via flyers and word-of-mouth invitations during undergraduate psychology lectures at Ulster University. Considering the experiment length (approximately 7 hours), participants were compensated for their time at a rate roughly equivalent to the minimum wage (£100 [US $133] for attending both sessions). All study data collected were anonymized at source with the use of participant ID codes. A spreadsheet (Microsoft Excel; Microsoft Corporation) linking participant ID codes with actual identities was maintained by the principal investigator. Participants were made aware that they could withdraw from the study at any time, and the means to do so was explained.

### Participants

Thirty healthy young adults (20 females and 10 males; aged 18-44 years, mean age 23 years, SD 6.8 years) were recruited from the student population at Ulster University. The sample size was determined using G*Power [39], with reference to previously published effect sizes from experiments that examined the effect of alcohol on similar cognitive tests [21,30]. Falleti et al [21] reported an effect size of *d*=0.6 in an N-back task following a similar level of alcohol consumption. Setting power at 0.8 and α at *P*=.05, the calculated sample size was N=32. Peterson et al [30] reported an effect size of *d*=0.3 on a delayed paired association task after alcohol consumption. With power set at 0.8 and α at *P*=.05, the calculated sample size was N=22.

Participants were screened as part of the online recruitment process. Exclusion criteria were pregnancy, self-reported current use of recreational or medicinal drugs, tobacco use, or heavy caffeine consumption (defined for this study as ≥3 large cups of coffee per day or the equivalent). The drinking status of applicants was determined using the Alcohol Use Disorders Identification Test (AUDIT; [40]), a 10-item questionnaire administered online. Responses from applicants scoring between 8 and 14 were reviewed by the principal investigator before inclusion (based on recommendations for identifying the risk of hazardous drinking behavior in the general population [41]), while applicants scoring 15 or above were excluded without review (higher cutoffs for identifying alcohol use disorders are recommended in student populations, particularly in the United Kingdom [42,43]). Five applicants were excluded based on their AUDIT responses/scores, and 1 was excluded due to self-reported recreational drug use.

### Benchmark Cognitive Tests

#### Overview

Tests were selected based on their relevance to cognitive dysfunction in the context of neurodegenerative and psychiatric disorders, as well as their established use in standard clinical testing batteries used in cognitive research and clinical practice. The targeted cognitive domains included episodic memory, executive function, psychomotor speed, and working memory.

#### Digit-Symbol Substitution (Coding) Test (DSST)

“The DSST is a paper-and-pencil cognitive test presented on a single sheet of paper that requires a subject to match symbols to numbers according to a key located on the top of the page. The subject copies the symbol into spaces below a row of numbers. The number of correct symbols within the allowed time...constitutes the score.” [44]. The WAIS-IV instructions, specifying a 120-second time limit, were used [45].

A license was obtained from the test publisher (Pearson) to modify the DSST for repeated administration in this study. Eighteen test variants were created by scrambling the key pairings, altering the order of rows on each page, and reversing the sequence of number prompts within individual rows. Tests were hand-scored independently by 2 researchers (RLQ and NK), who verified each other’s work. While alternate test forms can mitigate practice effects associated with learning the key pairings, some long-term benefits of repeated exposure have still been reported [46]. The DSST assesses a range of cognitive functions, including psychomotor speed, attention, planning, working memory, associative memory, and the executive functions of planning and strategizing [44].

#### Cambridge Neuropsychological Test Automated Battery (CANTAB) Paired Associates Learning (PAL)

The CANTAB PAL is a digital test of associative learning and episodic memory, extensively validated in mild cognitive impairment and dementia as a sensitive measure of hippocampal integrity [47]. The task requires participants to remember the locations of various abstract shapes presented in boxes around the edges of the screen. PAL can be administered in a browser window or on an iPad (Apple Inc) [48]. Both delivery methods were used in this study: the browser-based version during familiarization and the iPad version in the laboratory. The test provider (Cambridge Cognition) recommends 2 primary metrics: PAL Total Errors Adjusted (PALTEA28) and PAL First Attempt Memory Score (PALFAMS). PALTEA28 is typically selected as the primary outcome measure in studies investigating cognitive impairment, as it correlates well with other task metrics and is sensitive to early dementia [49]; therefore, it is the metric reported in the text here. PALFAMS is also reported in the multimedia appendices, where relevant. PALTEA28 is a negatively scored metric, with lower scores indicating fewer errors. In this context, “adjusted” refers to the estimation of error rates for later stages of the task that the participant may not have reached. This adjustment allows for meaningful score comparisons between participants who completed all levels and those who only completed the earlier stages.

#### Verbal Paired Associates—Part I (VPA-I)

The VPA subtest from the Wechsler Memory Scale version 4 (WMS-IV; [50]) is a verbal measure of episodic memory, administered by a trained experimenter. During the encoding stage, the experimenter presents word pairs to the participant. In the immediate recall stage, the experimenter provides the first word of each pair, and the participant is asked to recall the corresponding second word from memory. VPA-II (delayed recall and recognition) was not administered in this study due to time constraints. As the VPA does not include multiple forms, it was not used to assess the effect of alcohol; however, it was included to support the evaluation of convergent validity for the Memory Match task (Cumulus visual associative learning). The version used was the Adult Battery (for ages 16-69 years), with a maximum raw score of 56 words correctly recalled.

### The Cumulus Platform

#### The Cumulus Tablet App

**Figure 1:**
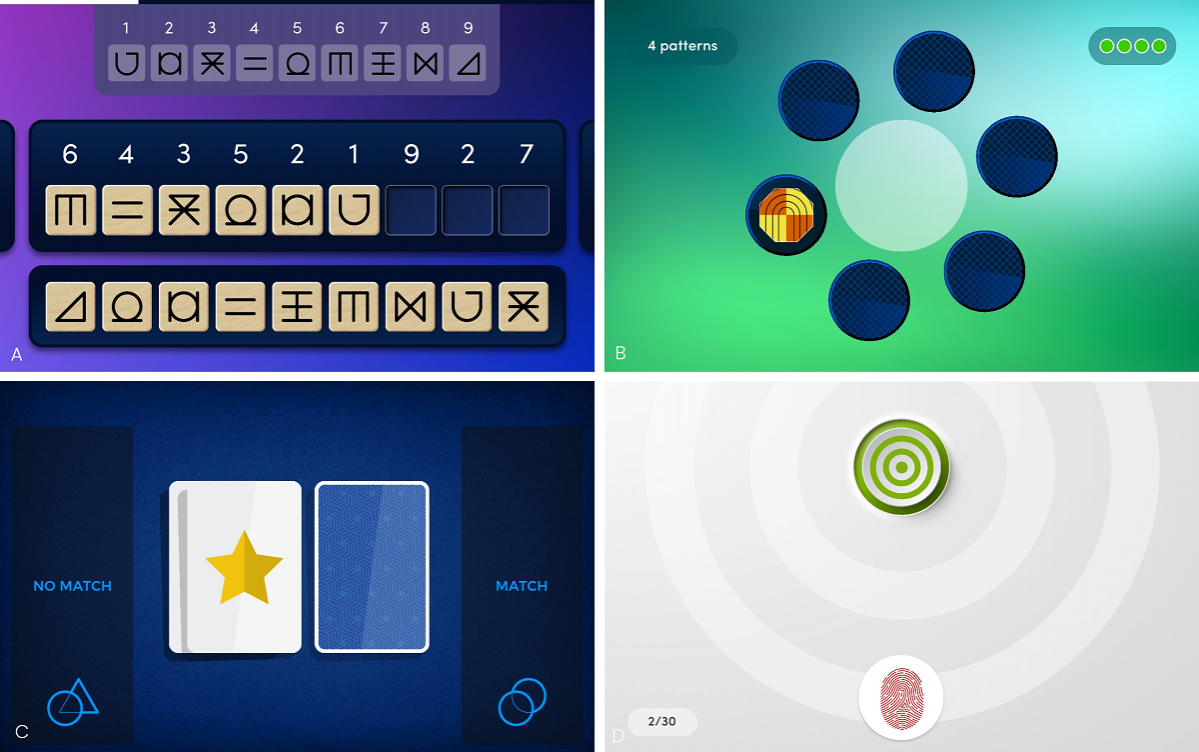
Task screenshots from the Cumulus mobile app. (A) “Symbol Swap” DSST analog; (B) “Memory Match” visual associative learning task; (C) “Double-Take” N-back; and (D) “Rapid Response” simple reaction time test. DSST: Digit Symbol Substitution Task.

**Figure 2:**
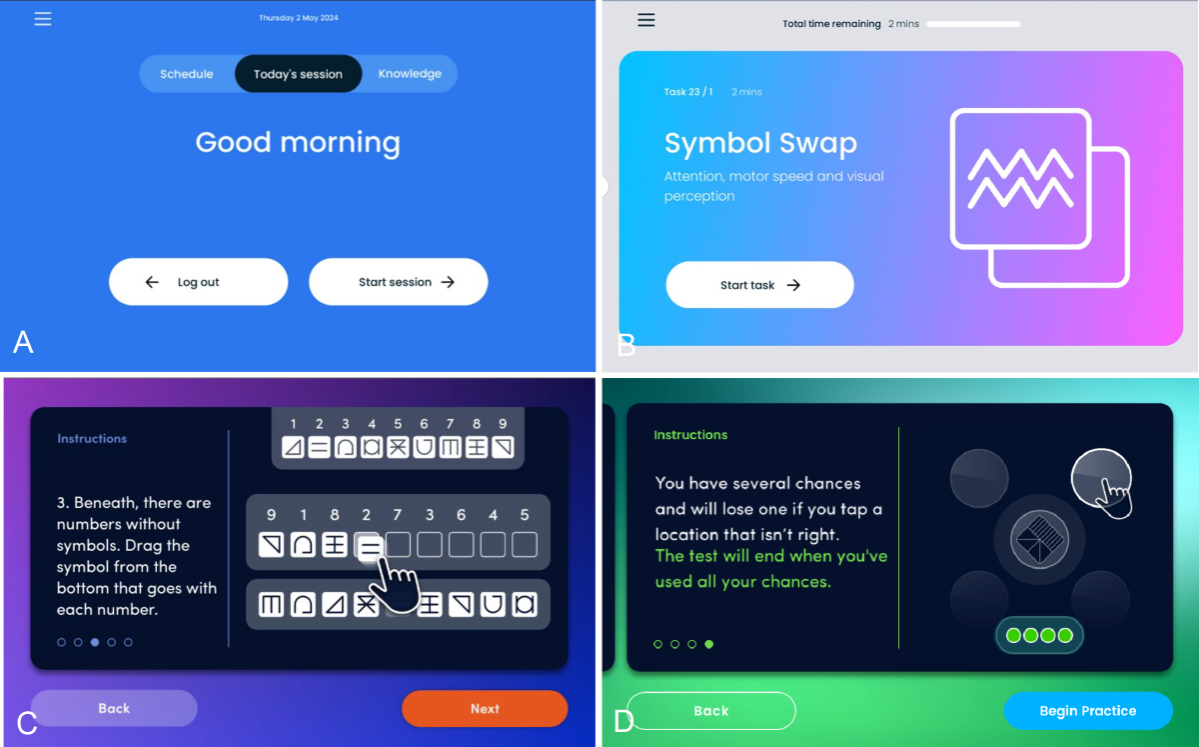
Screenshots of the tablet app, including nontask elements. (A) Post-log-in landing screen; (B) task list view, showing the current/next task to be completed; (C) 1 of 5 instructions screens before Symbol Swap; (D) 1 of 4 instructions screens before Memory Match.

The Cumulus app was developed for ease of use by individuals with cognitive impairment, such as older adults who may exhibit early symptoms of dementia. It features simple step-by-step instructions, task demonstrations, and a clear visual style. The 4 tasks deployed in this study were designed in consultation with a panel (n=10) of dementia experts, including patients, carers, and professionals. Panel members provided ongoing advice and feedback on visual clarity, instructional language, and overall task suitability throughout the development process. For example visuals, see Figures 1 and 2.

Tasks were administered on Lenovo TB-8505X tablets as a series of activities following a programmable schedule. Data were automatically uploaded to secure servers at the end of each session via a 4G mobile router. The app can be used in conjunction with a proprietary EEG headset, which was not included in this study [16].

#### Battery of Cumulus Tasks

##### Cumulus DSST Analog “Symbol Swap”

Designed to impose a similar cognitive demand as the symbol coding test (DSST) included in the WAIS [45], this task requires the participant to provide symbols in response to randomly ordered numbered prompts, according to a translation key at the top of the workspace. There are 9 digits (1-9) and 9 symbols, and the participant has 90 seconds to match as many symbols as possible. The 90-second time limit was chosen to align with the original WAIS, as task brevity was preferred over potential, but uncertain, gains in sensitivity. Of the 9 symbols, 2 pairs always resemble each other (lures) to increase difficulty. A very large number of alternate forms (>60 million) are available for repeat administrations. The primary outcome measure is the number of correct responses within the time limit. Like the original paper DSST, Symbol Swap assesses a range of cognitive functions, including psychomotor speed, attention, planning, working memory, associative memory, and the executive functions of planning and strategizing [44]. Including instructions and test-taking time, the total duration of Symbol Swap is approximately 3 minutes.

##### Cumulus Visual Associative Learning Task “Memory Match”

The hippocampus is functionally involved in spatial and episodic memory. Its degeneration contributes to the forgetfulness associated with the early stages of AD [51], while reduced hippocampal neurogenesis has been linked to impaired recollection in unipolar depression and mood-related disorders [52,53]. Memory Match targets these functions by asking participants to learn and remember an increasing number of abstract patterns at different locations on the screen. The task consists of several “encoding” and “recall” stages, which become progressively more difficult until the participant’s “lives” (ie, chances to make an error and still continue) are exhausted. Four lives are available per session.

During each encoding phase, the participant must commit to memory the location of novel patterns, distributed across 6 on-screen locations. At the recall stage, the participant must recall these novel patterns, along with all patterns presented in previous levels. Patterns appear in the center of the screen, and responses are made by tapping on a location. Feedback (correct vs incorrect, with a correction for incorrect responses) is provided for each response. The task is designed to be accessible at all levels of ability, starting with gradual increases in difficulty and becoming more challenging in later levels. The primary outcome measure is the number of correct responses before the task ends due to the depletion of lives. Lives do not regenerate. As a result of the lives mechanic, task duration is variable, ranging from 4 to 15 minutes.

To prevent stimulus-related practice effects, a different set of patterns is used for each administration. Thirty unique sets of 49 patterns are currently available. In each session, 1 set is selected at random from those that the user has not yet seen.

##### Cumulus N-Back Task “Double-Take”

Double-Take is a match/nonmatch N-back paradigm in which the participant must compare the current stimulus with one presented “N” items earlier, or “back.” This task targets working memory and executive function, which decline with age and more precipitously in pathological aging [54,55]. Working memory deficits are also a common feature of schizophrenia [56] and MDD [57].

Thirty trials are delivered at 1-back, followed by 30 trials at 2-back, with a self-paced break between them. Responses are made by tapping “match” or “no match” on either side of the screen. The trial sequence varies between administrations (ie, the order of stimuli and the occurrence of match/nonmatch trials are shuffled), with approximately one-third of the trials being “match” trials. The main outcome measures are accuracy and reaction time, by trial type. These measures assess working memory (which includes executive functions such as attention and sequencing) and psychomotor speed, respectively—though only the former is reported in this study. Strong practice effects in healthy participants have previously been reported on N-back tasks, which appear to be primarily procedural in nature [58]. The duration of the Double-Take is approximately 4 minutes.

##### Cumulus Simple Reaction Time Task “Rapid Response”

Reaction time is a direct measure of processing speed and can serve as a highly sensitive (though nonspecific) assay of neurocognitive functioning [38,59]. This task consists of a “ready button” and a distal target. During 10 practice trials with on-screen instructions, participants are guided to place and hold their finger on the ready button and wait for the target to turn green, signaling that it should be tapped as quickly as possible.

There are 30 test trials, with the main outcome measures being reaction time and the SD of reaction time. This task is identical in every administration (though the practice trial block can be skipped starting from the second administration). The duration of Rapid Response is approximately 3 minutes.

### BAC Measurement and Subjective Measure of Intoxication

An electrochemical breathalyzer (Dräger Alcotest 6000; [60]) was used throughout the experiment to measure BAC%. This device is approved for police use in the European Union and uses technology that has been found to correlate highly with blood sampling in laboratory conditions [61].

A Visual Analog Scale (VAS) for subjective intoxication [36] was administered throughout the experiment to assess the participant’s subjective intoxication level. This consists of a plain horizontal line with “Least intoxicated ever felt in life” on the left and “Most intoxicated ever felt in life” on the right. The prompt asks “How intoxicated do you feel right now?” Respondents are asked to place a mark on the line representing their current feeling of intoxication, and its position is later measured using a ruler to extract a value.

### Study Design and Procedure

#### Study Design

**Figure 3:**
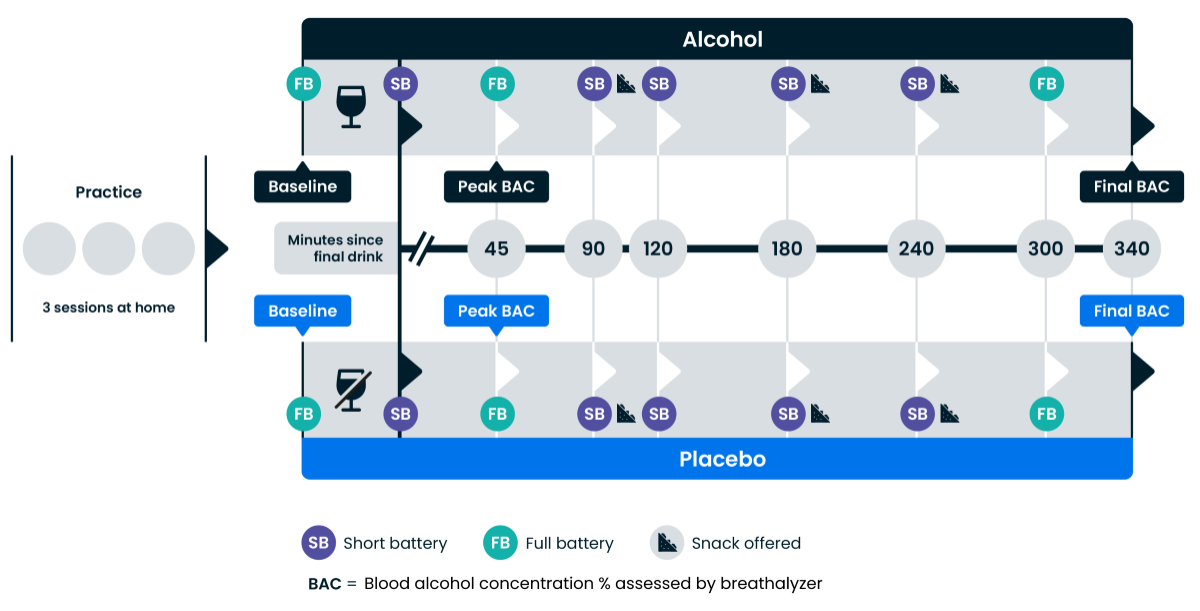
Schematic diagram of the study protocol. Every participant completed both arms on separate days (order counterbalanced).

The experiment follows a repeated-measures, placebo-controlled (single-blind) crossover within-participant design (see Figure 3). The order of treatment was counterbalanced, such that half of the participants (n=15) received alcohol in their first session and a placebo in their second. Participants attended the laboratory on 4 occasions: for their initial study visit, followed by 2 testing visits during which they alternatively received either an alcoholic drink or a placebo.

#### Alcohol Dosing and Placebo

A participant-specific alcohol dose was calculated for each individual using an online BAC calculator [62] and a measure of body weight taken during the initial study visit. The alcoholic drink administered during the testing sessions consisted of 40% ABV vodka mixed with orange juice, while the placebo consisted of orange juice only. To ensure proper blinding of participants, a mist of vodka was applied over the top of every glass, regardless of its content. The initial dose was split into 3 servings, with participants instructed to drink 1 serving every 10 minutes. Forty-five minutes after the third serving, BAC% was measured using the breathalyzer. The target range was 0.08%-0.1% BAC. Following the procedure of [36], if a participant did not reach 0.08%, they were given a top-up serving of alcohol, based on the BAC% target delta.

#### Initial Study Visit

Participants first attended a meeting with the researcher, during which a screening questionnaire was completed, and all tasks used in the study were demonstrated. Some practice effects on all tests were assumed, so massed-practice familiarization sessions were conducted before the in-laboratory sessions to overcome these effects and generate data for direct measurement of any practice effects.

- Following advice from the test provider (Cambridge Cognition), who has previously characterized practice effects on PAL in healthy participants, the CANTAB PAL test was completed once before the first in-laboratory session. Participants were provided with a unique link to access the test at home via a web browser.
- The paper DSST was completed twice before the first laboratory session, supervised by a researcher (RLQ or NK). Alternate forms were used for each administration, including the practice runs.
- The 4 Cumulus tasks were completed once during the meeting and 2 more times remotely. Participants were loaned a tablet to perform the second and third runs at their convenience.
- A practice run of VPA was not possible due to the unavailability of alternate forms of the test (ie, additional validated word stimuli).

#### Testing Sessions

Completion of all practice tests was verified on the morning of the first testing session. If any records were missing, the respective practice sessions were conducted in person.

Participants were asked to refrain from using caffeine on the day of the testing sessions and from consuming alcohol the night before the study. They were also asked to have a “good night’s sleep” the night before, if possible, and to have a normal breakfast.

There were 8 measurement points (including the baseline) during each testing day, which lasted approximately 6 hours. Because of time constraints, not all tests were administered at every measurement point. Two batteries were constructed:

- Full battery (approximately 37 minutes): a full battery of Cumulus tasks (Symbol Swap, Rapid Response, Memory Match, and Double-Take); paper DSST; CANTAB PAL; VPA-I (baseline only); subjective intoxication VAS; and breathalyzer (2 times: at the start and at the end of the measurement point).
- Short battery (approximately 13 minutes): a limited battery of Cumulus tasks (Symbol Swap and Rapid Response), paper DSST, subjective intoxication VAS, and breathalyzer (2 times: at the start and at the end of the measurement point).

The tests selected for the short battery were those with the shortest duration.

Testing on the in-laboratory days proceeded as follows:

For each measurement point, the order of the Cumulus tasks was counterbalanced across participants to control for order and fatigue effects. For the full battery, 4 arrangements of the task order were available: [A: 1, 2, 3, 4]; [B: 2, 3, 4, 1]; [C: 3, 4, 1, 2]; [D: 4, 1, 2, 3]; 15 participants (50%) received order A during their initial session, and the other 15 (50%) received order C. Thereafter, the arrangements were cycled through in the same order outlined here (A-B-C-D). For the short battery, which contains 2 tasks and thus offers only 2 arrangements (1, 2 or 2, 1), half of the participants (n=15, 50%) began with the [1, 2] order and alternated thereafter. Non-Cumulus tests were also counterbalanced between participants by placing them alternately before or after the block of Cumulus tasks at each measurement point.

Baseline measurement consisted of the full battery, including VPA-I. Between baseline and the next measurement point, alcohol or placebo was administered. As stated above, the initial dose was split into 3 servings. Immediately after the consumption of the third serving, a short battery was administered to obtain a measurement of performance while BAC was building, but before the peak (Figure 2). This measurement point is referred to as “initial dose” in all subsequent tables, plots, and analyses. Intoxication was measured at this time point using the breathalyzer and VAS, though the breathalyzer measurements were not valid due to the proximity to drinking.

The “+45-minute” assessment occurred 45 minutes after the final serving of alcohol/placebo. In the alcohol condition, this refers to 45 minutes after the final top-up dose (if applicable). Because of the long duration of assessment at some measurement points, and the assumption that BAC% might change over such a period, breathalyzer measurements were taken at the start and end of each measurement point, with the results averaged.

### Data Analysis

#### Analysis of Practice Effects

Practice effects on each Cumulus task were assessed using a Wilcoxon signed rank test between each time point in the prestudy period, comparing each task administration with the next. Holm-Bonferroni correction was applied to *P* values to control the type 1 error rate. Descriptive statistics, along with *Z* and adjusted *P* values, are reported, with α set at .05.

#### Effect of Alcohol on Cognitive Functions

The primary alcohol analysis examined the effect of change over time within the study session and how it was influenced by condition (ie, alcohol dosing). For each task, a linear mixed-effects model was used to assess the impact of alcohol on task performance over time. The same model arrangement was used for analyzing the primary outcome variable of each cognitive task. Covariates including age, sex, and the chronological session number (indicating on which of the 2 days a given assessment occurred) were added as fixed effects to the linear mixed models. This was done to test for longer-term between-session practice effects independent of treatment. A random effect of participants was also included to account for individual variation. Model-wise Holm-Bonferroni adjustment was applied to *P* values to control the type 1 error rate, with α set at .05. The normality of the residual distribution was assessed by visual inspection. Subsequently, performance data for Rapid Response and Double-Take were 10log(x) transformed before modeling because the model residuals were not normally distributed in the metrics’ linear scale.

#### Evaluation of the Construct Validity of the Cumulus Tasks

Convergent and construct validity of the Cumulus tasks were assessed using Spearman rank correlations. Correlations were anticipated based on the overlap between the cognitive domains targeted by the various tasks. Correlations were planned across the following cognitive domains:

- Episodic Memory domain: Memory Match (total number recalled), CANTAB PAL (total errors adjusted), and VPA-I (memory score); and
- Executive function/general performance domain: Symbol Swap (total correct responses), paper DSST (total correct responses), and Rapid Response (mean reaction time).

Correlations were performed on data obtained from the +45-minute time point on the alcohol day only (peak BAC%), due to the expectation of some ceiling effects on nonalcohol measurements. VPA-I correlations are an exception, as this task was only performed once, at baseline. Note that analog comparator tests directly matching the cognitive demands of Double-Take (Cumulus N-back) and Rapid Response (Cumulus simple reaction time) were not included for comparison in this study, due to the lack of sufficiently well-established gold-standard implementations for these tasks in the field. Additionally, Double-Take is not evaluated for construct validity against other tasks in the study, due to the uniquely heavy reliance of N-back performance on working memory information sequencing/updating, and its much lighter reliance on the cognitive functions most prominently linking the other tasks (episodic memory and psychomotor speed).

## Results

### Digital Data Integrity

As described previously, task ordering and data upload were handled automatically by the Cumulus app and backend. Technical issues related to Wi-Fi availability resulted in the loss of 1 set of task data—an administration of Symbol Swap (Cumulus DSST) at the +300-minute time point, for 1 participant. All other task data were uploaded successfully after the provision of a 4G mobile router. In summary, performance data for 1679 of 1680 administered Cumulus tasks were collected.

### Practice Effects for Cumulus Tasks

Three practice sessions using the Cumulus platform were completed by all participants before their first in-laboratory assessment. Descriptive statistics are shown in Table 1. For Symbol Swap, a significant difference was found between the first and second practice sessions for total correct responses: *Z*=51; *P*_(corrected)_=.045. No other significant practice effects were observed for task performance metrics during the prestudy period. All Wilcoxon test results (including corrected and uncorrected *P* values) can be found in Multimedia Appendix 1.

**Table 1:** Descriptive statistics from cognitive tasks completed during practice, in the days before the study period. The Cumulus tasks comprise Symbol Swap (digital DSST), Memory Match (visual associative learning), Rapid Response (simple reaction time), and Double-Take (N-back).

Task metric	Practice 1, mean (SD)	Practice 2, mean (SD)	Practice 3, mean (SD)
Symbol Swap total correct	31.9 (6.8)	35.1 (6.6)	35.8 (7.8)
Memory Match total correct	24.8 (12.5)	26.4 (16.7)	27.4 (16.1)
Rapid Response (mean reaction time) (ms)	456.0 (70.2)	449.0 (66.5)	439.2 (69.8)
Rapid Response (SD reaction time) (ms)	142.7 (163.2)	95.9 (46.0)	85.9 (60.2)
Double-Take (1-back) accuracy on match trials (%)	92.3 (15.8)	95.2 (7.0)	98.6 (4.7)
Double-Take (2-back) accuracy on match trials (%)	72.4 (21.6)	79.6 (21.9)	84.5 (13.6)
Paper DSST total correct	77.9 (18.1)	77.2 (19.9)	N/A^a^
PAL^b^ First Attempt Memory Score (PALFAMS28)	14.4 (3.3)	N/A	N/A
PAL Total Errors Adjusted (PALTEA28)	9.5 (9.2)	N/A	N/A

^a^N/A: not applicable.

^b^PAL: Paired Associates Learning.

### Alcohol Consumption

**Figure 4:**
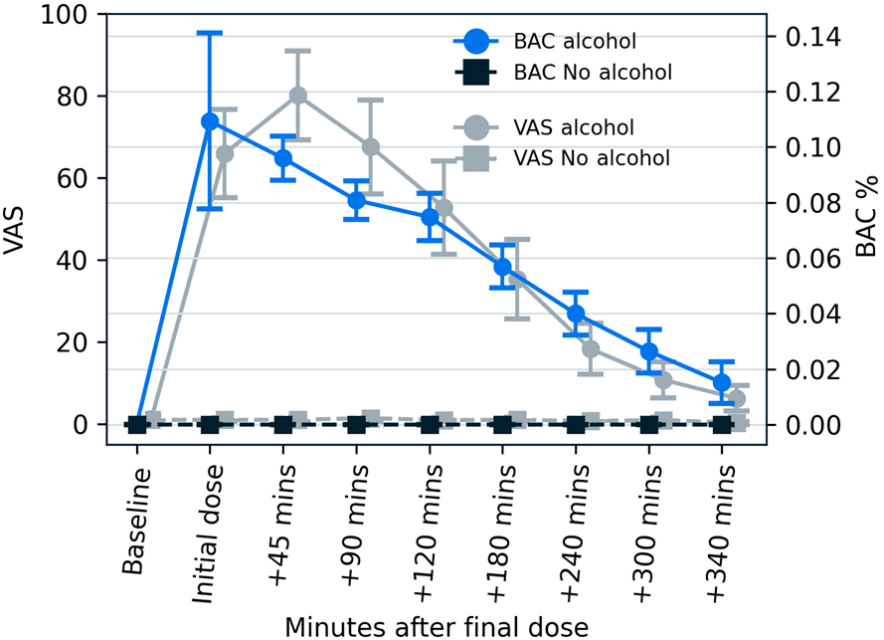
Time plot showing BAC% and VAS self-reported intoxication over time during the study period for the alcohol and no-alcohol sessions. BAC: blood alcohol concentration; VAS: Visual Analog Scale.

Top-up doses were required for 22 of 30 alcohol sessions, possibly indicating limitations in the online calculator used or differences between the current sample and the calculator’s calibration data (eg, calculator-assumed fasting, relative aldehyde dehydrogenase upregulation in participants, or similar factors). The total alcohol administered to reach the target BAC per participant ranged from 125 to 425 mL of vodka. The research team reported that a significant proportion of participants could distinguish between the alcohol and placebo conditions, as supported by the subjective intoxication ratings shown in Figure 4.

The mean score on the alcohol use questionnaire (AUDIT) was 7.03 (SD 2.71).

Subjective intoxication (VAS) peaked at +45 minutes postalcohol and declined thereafter (see Figure 4). BAC% followed a similar pattern but appeared more variable (inflated) at the “Initial dose” time point, likely due to residual alcohol in the mouth and throat when measurements were taken, which may have confounded these measurements.

### Effect of Alcohol on Performance

**Figure 5:**
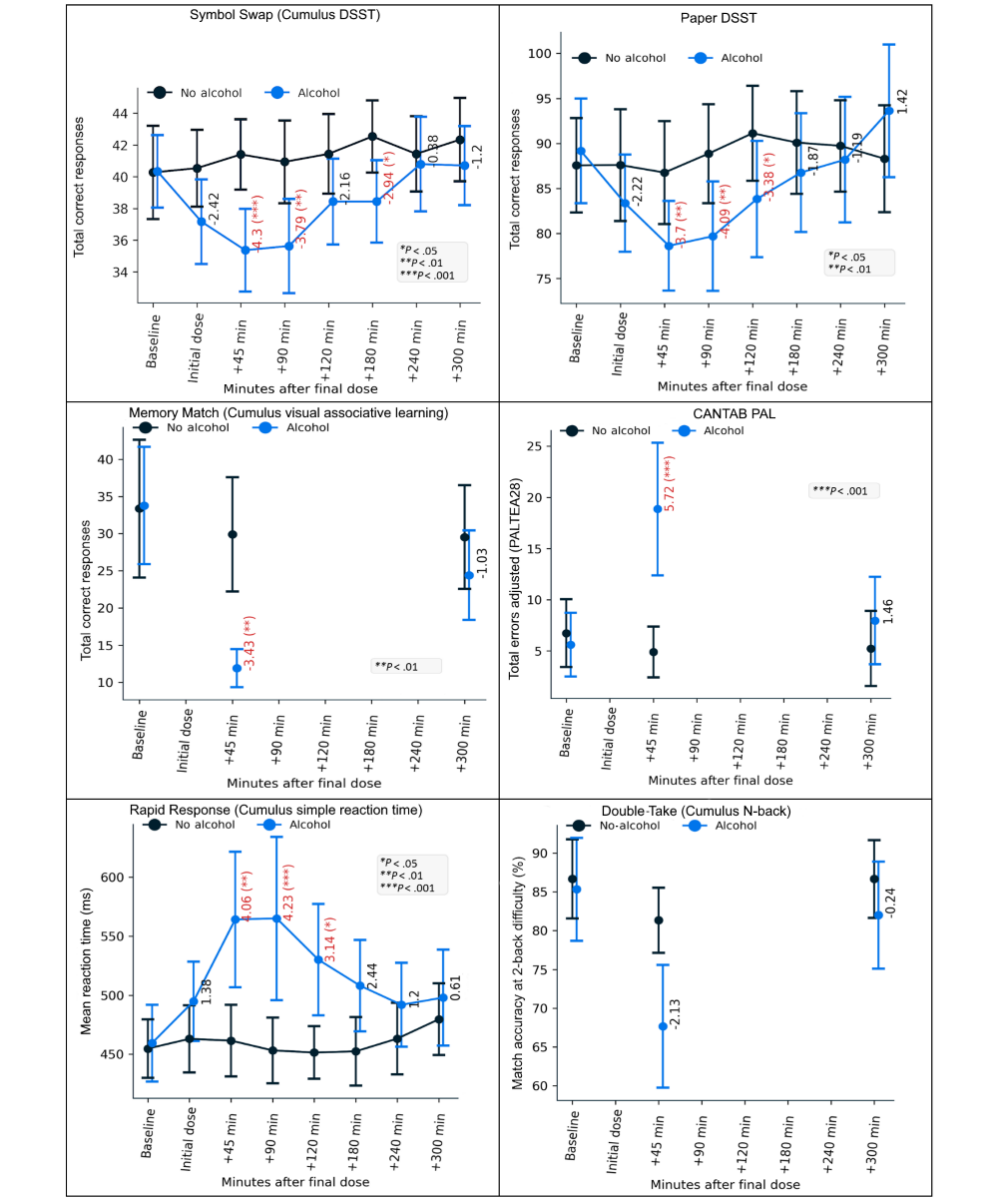
Time plots showing alcohol/placebo performance curves for the primary metrics in each of the 6 tasks. Error bars are 95% CIs. *t* values are shown for the interaction of session type (alcohol/placebo) and session time point, with significant interactions highlighted (Holm-Bonferroni correction applied). Note that while raw task metrics are plotted here, *t* values and significance were derived from 10log(x)-transformed data for Rapid Response (Cumulus simple reaction time) and Double-Take (Cumulus N-back). CANTAB: Cambridge Neuropsychological Test Automated Battery; DSST: Digit Symbol Substitution Task; PAL: Paired Associates Learning; PALTEA: Paired Associates Learning Total Errors Adjusted.

Alcohol impaired performance on all cognitive assessments examined, except for Double-Take (Cumulus N-back—see Table 2; Figure 5). The main results of the linear models (with Holm-Bonferroni–corrected *P* values; significance criterion=.05) are summarized here for brevity, with the full model output (including raw *P* values) for every metric tested provided in Multimedia Appendix 2.

For Symbol Swap (Cumulus DSST), performance on the task (assessed via the total number of correct responses within the time limit) was impaired by alcohol at time points +45 minutes (*P*<.001); +90 minutes (*P*=.003); and +180 minutes (*P* = 0.048). Additionally, there was an effect of visit number (*P*<.001), indicating a between-day practice effect, with an average improvement of approximately 2.9 correct responses (β=2.912). For Memory Match (Cumulus visual associative learning), memory performance (measured by the total number of items correctly recalled) was impaired by alcohol at time point +45 minutes (*P*=.005). For Rapid Response (Cumulus simple reaction time), the mean reaction time was slowed by alcohol at time points +45 minutes (*P*=.001), +90 minutes (*P*<.001), and +120 minutes (*P*=.03). Similarly, the SD of reaction time was greater in the presence of alcohol at time points +90 minutes (*P*<.001), +120 minutes (*P*=.01), and +180 minutes (*P*=.01). For Double-Take (Cumulus N-back), accuracy on match trials at 1-back difficulty was impaired by alcohol at time point +45 minutes (*P*=.02). Accuracy on match trials at 2-back difficulty was not affected by alcohol. However, overall accuracy (match and nonmatch trials combined) at 2-back was reduced by alcohol at +45 minutes (*P=*.03).

For the paper-based DSST, performance was impaired by alcohol at time points +45 minutes (*P*=.004), +90 minutes (*P*=.001), and +120 minutes (*P*=.01). The effect of visit number was also statistically significant (*P*<.001), indicating the presence of a between-days practice effect. For the CANTAB PAL (metric: total errors adjusted), alcohol impaired performance at time point +45 minutes (*P*<.001). Similarly, alcohol also affected performance on the alternative PAL metric, First Attempt Memory Score (PALFAMS28), at time point +45 minutes (*P*<.001).

**Table 2:** Descriptive statistics from all cognitive tasks across all measurement time points in the study period, in both alcohol and placebo conditions. Cumulus tasks include Symbol Swap (digital DSST^a^), Memory Match (visual associative learning), Rapid Response (simple reaction time), and Double-Take (N-back). Verbal Paired Associates—Part I was administered during the first laboratory session only, regardless of the condition assignment for that day.

Task metric and session			Baseline, mean (SD)	Initial dose, mean (SD)		+45 minutes, mean (SD)		+90 minutes, mean (SD)		+120 minutes, mean (SD)		+180 minutes, mean (SD)		+240 minutes, mean (SD)		+300 minutes, mean (SD)
**Symbol Swap (Cumulus DSST) total correct**																
	Alcohol	40.3 (6.3)		37.2 (7.3)	35.4 (7.1		35.6 (8.1)		38.4 (7.4)		38.4 (7.1)		40.8 (8.1)		40.7 (6.8)	
	No alcohol	40.3 (8.0)		40.5 (6.6)	41.4 (6.1		40.9 (7.1)		41.4 (6.9)		42.5 (6.2)		41.4 (6.5)		42.3 (7.2)	
**Memory Match (Cumulus visual associative learning) total correct**																
	Alcohol	33.8 (21.6)		N/A^b^	11.9 (7.0		N/A		N/A		N/A		N/A		24.4 (16.5)	
	No alcohol	33.4 (25.4)		N/A	29.9 (21.0		N/A		N/A		N/A		N/A		29.5 (19.1)	
**Rapid Response (Cumulus simple reaction time; mean reaction time [ms])**																
	Alcohol	459.4 (88.9)		494.8 (92.3)	564.2 (157.6		565.1 (189.7)		530.1 (129.1)		508.1 (105.9)		491.8 (97.4)		498.0 (111.4)	
	No alcohol	454.7 (67.7)		463.0 (77.9)	461.5 (83.4		453.2 (76.3)		451.4 (61.0)		452.4 (79.4)		463.2 (83.1)		479.6 (83.2)	
**Rapid Response (Cumulus simple reaction time; SD reaction time [ms])**																
	Alcohol	91.1 (68.4)		104.3 (66.5)	189.6 (252.4		243.1 (253.7)		140.4 (108.1)		140.1 (111.6)		130.3 (116.4)		124.5 (118.7)	
	No alcohol	106.7 (70.9)		86.1 (39.5)	97.8 (87.2)		92.6 (83.9)		80.2 (56.6)		76.5 (40.9)		84.7 (42.5)		113.6 (86.3)	
**Double-Take (Cumulus N-back) accuracy on match trials at 1-back (%)**																
	Alcohol	97.0 (5.3)		N/A	89.3 (8.9)		N/A		N/A		N/A		N/A		94.0 (8.8)	
	No alcohol	98.3 (4.5)		N/A	98.3 (3.7)		N/A		N/A		N/A		N/A		95.7 (7.2)	
**Double-Take (Cumulus N-back) accuracy on match trials at 2-back (%)**																
	Alcohol	85.3 (18.2)		N/A	67.7 (21.7)		N/A		N/A		N/A		N/A		82.0 (18.9)	
	No alcohol	86.7 (14.0)		N/A	81.3 (11.5)		N/A		N/A		N/A		N/A		86.7 (13.7)	
**Paper DSST total correct**																
	Alcohol	89.2 (15.9)		83.4 (14.8)	78.6 (13.6)		79.7 (16.6)		83.8 (17.7)		86.8 (18.0)		88.2 (19.1)		93.6 (20.2)	
	No alcohol	87.6 (14.4)		87.6 (17.0)	86.8 (15.7)		88.9 (15.1)		91.1 (14.5)		90.1 (15.6)		89.7 (13.9)		88.3 (16.2)	
**PAL^c^ First Attempt Memory Score (PALFAMS28)**																
	Alcohol	16.1 (3.4)		N/A	11.8 (4.8)		N/A		N/A		N/A		N/A		15.3 (3.9)	
	No alcohol	15.5 (3.8)		N/A	16.4 (3.3)		N/A		N/A		N/A		N/A		17.0 (3.2)	
**PAL Total Errors Adjusted (PALTEA28)**																
	Alcohol	5.6 (8.5)		N/A	18.9 (17.7)		N/A		N/A		N/A		N/A		8.0 (11.7)	
	No alcohol	6.7 (9.1)		N/A	4.9 (6.8)		N/A		N/A		N/A		N/A		5.2 (10.0)	
**Verbal Paired Associates—Part I**																
	First session	35.7 (7.5)		N/A	N/A		N/A		N/A		N/A		N/A		N/A	

^a^DSST: Digit Symbol Substitution Task.

^b^N/A: not applicable.

^c^PAL: Paired Associates Learning.

### Convergent Validity of Cumulus Versus Benchmark Tests

Correlational analyses were conducted using data from +45 minutes on the alcohol day (peak intoxication) to capture a meaningful spread in performance scores. VPA-I correlations are the exception, as this task was only performed at baseline and is therefore correlated with other baseline assessments. Raw *P* values are reported, as each test is considered a separate family of statistical inferences.

Spearman rank correlations were performed on task metrics within the Episodic Memory domain, revealing a significant negative relationship between total correct responses on Memory Match (Cumulus visual associative learning) and total errors adjusted on the CANTAB PAL (*r*_28_=–0.61; *P*<.001). Performance on VPA-I did not correlate with performance on either of the other 2 episodic memory tasks (at baseline): VPA-I versus PAL total errors adjusted (*r*_28_=–0.3; *P*=.11) and VPA-I versus Memory Match total correct (*r*_28_=0.27; *P*=.15). Note that a higher score on PAL total errors adjusted corresponds to more errors, so a negative correlation with the other (positively scored) task metrics is expected.

In the Executive Function/General Performance domain, total correct responses on Symbol Swap (Cumulus DSST) were positively correlated with total correct responses on the paper DSST (*r*_28_=0.65; *P*<.001). Additionally, total correct responses on Symbol Swap (Cumulus DSST) were negatively correlated with the SD of reaction time on Rapid Response (Cumulus simple reaction time) (*r*_28_=–0.38; *P*=.04). Performance on the paper DSST (total correct) was negatively correlated with both the mean reaction time (*r*_28_=–0.47; *P*=.009) and the SD of reaction time (*r*_28_=–0.46; *P*=.01) on Rapid Response.

## Discussion

This study demonstrates that cognitive performance can be tracked over time using the current digital cognitive battery, in line with Falleti et al [21]. The tasks in this battery closely correspond to their benchmark equivalents (where applicable) and can be administered through an integrated, automated platform that is deployable remotely with minimal administrative effort [25]. Practice effects were observed on 1 digital task, but these were minimal compared with the cognitive changes measured, and were consistent with practice effects on its paper counterpart (DSST). Scalable, repeatable, and low-burden testing platforms are crucial for evaluating the impact of disease-modifying interventions in psychiatry and neurodegeneration. The cognitive battery examined in this study could be part of the solution, whether used in the clinic or autonomously at home by patients with cognitive and motor impairments.

Along with the Cumulus tasks, the 2 benchmark tests (paper DSST and CANTAB PAL) also showed clear sensitivity to alcohol at the peak intoxication time point (45 minutes postdose), with the paper DSST effect lasting up to 120 minutes postdose. It is notable that the paper DSST showed impaired performance at 120 minutes, whereas Symbol Swap (the Cumulus digital alternative) did not. This could reflect data noise or insufficient statistical power, as mean differences were observed in Symbol Swap at +120 minutes, but the *P* value did not survive multiple comparisons correction (see Multimedia Appendix 2). For clinical meaningfulness, cognitive impairment 45 minutes postalcohol resulted in a reduction of 9.73 points (β=9.73) on the paper DSST, and a reduction of 5.76 points (β=5.76) on Symbol Swap (Cumulus DSST). These reductions are slightly larger than the “minimal clinically important difference” of around 4 on the SDMT specified by Borland [4] for cognitively healthy older adults. However, direct comparison is challenging due to differences in the response capture mechanism (eg, symbol writing vs letter writing vs screen swiping) and the characteristics of the groups studied, such as variability due to age, which may contribute to the observed differences. Alternatively, Jutten et al [63] reported a mean difference of 21.3 correct responses on a 90-second paper-based DSST (identical in format to the paper test used in this study) between a group of patients with mild cognitive impairment-to-mild-AD dementia and cognitively healthy controls of similar age. This comparison suggests that Borland’s benchmark for individual clinical change (around 4 points on the SDMT) and the changes observed in this study on similar tasks are much more subtle than the cognitive differences associated with the manifestation of AD dementia.

Broadly, we observed correlations during peak intoxication between the Cumulus tasks and well-validated benchmark equivalents designed to measure the same or overlapping cognitive functions (ie, within the “episodic memory domain” and “executive function/general performance domain”). For example, performance on Memory Match (Cumulus visual associative learning) was strongly associated with CANTAB PAL performance. This supports the hypothesis of a shared neurocognitive basis underlying both tasks, which require learning item-location pairs. This operationalizes “recollection memory,” which is impaired in the early stages of AD dementia due to hippocampal dysfunction [47]. Similarly, patients with MDD and mood-related disorders also experience impaired recollection, associated with abnormal hippocampal neurogenesis and volume [53,64]. VPA-I did *not* correlate with either the Cumulus memory task or the PAL benchmark task, despite clear functional overlap (learning paired material). VPA is a clinical tool primarily used to detect abnormal memory performance due to injury or illness (eg, neurodegeneration in older age) and may lack meaningful variance in this group of healthy, unimpaired undergraduate students (who were sober at the time). Measurement of VPA-I at peak intoxication or in a disease population could yield a different result. Alternatively, differences in the stimulus types to be learned across tasks (eg, abstract visual patterns in radial on-screen locations vs paired words), along with the distinct encoding strategies required [65], may explain why the tablet-based tasks (PAL and Memory Match) correlate with each other but not with VPA-I.

In the Executive Function/General Performance domain, a strong correlation was observed between the Cumulus DSST (Symbol Swap) and its comparator (paper DSST), reflecting substantial cognitive overlap in task demands. More moderate correlations were also found between both symbol-coding variants and Rapid Response, for both mean reaction time and SD metrics. This is consistent with the supposition that psychomotor speed accounts for some, but not all, of the variance in performance on symbol-coding tasks [44]. To summarize, the generally strong correlations [66], along with the corresponding sensitivity to alcohol-induced impairment, demonstrate that the Cumulus measures have concurrent validity and can be used in similar contexts to detect cognitive change.

A secondary aim of this study was to assess practice effects associated with repeated administration of the novel tasks. Practice effects are an important and often underappreciated feature of long-term neurocognitive monitoring, and can result in the underestimation of cognitive decline [17]. Limited evidence of practice effects was found during the prebaseline massed practice sessions—a significant improvement between practice sessions 1 and 2 for Symbol Swap (Cumulus DSST) was the only instance observed. Nevertheless, this should not be interpreted as evidence of an absence of practice effects on these tasks per se. For example, participants who are less comfortable with technology (eg, some older individuals) or who have reduced neuroplasticity might exhibit a different learning profile across administrations. Some tasks—namely, Symbol Swap and the paper-based DSST—also showed longer-term practice effects associated with the order of laboratory visits; that is, performance was, on average, slightly better on the second chronological day spent in the laboratory. This is consistent with the literature on symbol coding [46], though it remains to be seen how many sessions are required to fully extinguish practice effects on such tasks.

One Cumulus task, Double-Take—the digital “N-back” working memory task—was not sensitive to alcohol impairment at peak intoxication on the hypothesized end point, despite contrary expectations based on the literature [34,35]. Accuracy on “match” trials at the 2-back difficulty level was selected as the primary task metric, given the maximal demand these trials place on working memory systems. That is, the participant must positively identify that the item on-screen matches the one presented 2 positions earlier, relying on information held in working memory. Therefore, it was expected that this metric would be maximally sensitive to cognitive perturbation induced by alcohol consumption. Other task metrics—such as overall accuracy at the 2-back level, or match accuracy at 1-back—were found to be sensitive to alcohol at 45 minutes postdose in the current experiment, but are not reported in the main text, as they were not part of the primary hypothesis–driven results (see Multimedia Appendix 2). It is possible that the low number of match trials (10 per N-back level), and the resulting lack of granularity in the associated metric, contributed to the absence of a significant effect on the primary metric. One way to address this issue could be to increase the number of trials—though this might reduce participant compliance in a longitudinal at-home study, as the increased difficulty of 2-back makes it considerably less enjoyable than 1-back. However, it is encouraging that match trial accuracy at n=1 was sensitive to alcohol, as levels beyond 1 may be too challenging altogether for patients with substantial cognitive impairment.

A limitation of this study was the lack of comparison to well-established benchmark tests for Double-Take (Cumulus N-back) and Rapid Response (Cumulus simple reaction time). In the absence of a gold-standard archetype for these tasks—particularly given the substantial variability in N-back implementations—evaluating construct validity is challenging. Instead, validity may need to be inferred from demonstrated sensitivity and specificity to modulations of the underlying cognitive functions. This study demonstrates sensitivity to the relatively broad effects of alcohol, but further research is needed to address the remaining gaps. Another limitation of the study was the lack of control over the timing of massed practice sessions. Participants were allowed to complete these sessions over several days before their first laboratory visit, meaning that practice could have occurred in a single block, been spaced out, or completed early or late within the permitted window. It is possible that these differences led to more or less effective learning across individuals, thereby introducing variability into our analysis of practice effects. A more effective approach would have been to enforce a standardized timing protocol for practice sessions, allowing for better control over the rate and depth of learning [67]. Lastly, tools that more clearly define cognitive changes in clinically meaningful terms would have aided in the interpretation of the current results. For example, in addition to rating their perceived intoxication on a VAS, participants could have been asked whether they felt capable of competently performing certain daily activities (eg, driving or shopping).

Our findings on the effects of alcohol suggest that this digital cognitive battery is well-suited for high-density measurement of subtle cognitive changes over time in a laboratory setting. It may therefore be valuable in contexts where traditional neuropsychological testing would be prohibitively burdensome. In this study, 1679 individual cognitive tasks were self-administered by participants via tablet and scored automatically, with researcher involvement limited to scheduling the start of sessions. This arrangement could prove valuable in a clinical trial context, where the limitations of existing tools—such as poor repeatability, the high burden for both patients and staff, and the high within-participant variability—make detecting cognitive change particularly challenging [68].

The tasks in the battery were developed in collaboration with experts from the pharmaceutical industry, and this study was designed to validate them to standards that could support their use in clinical trials of new therapies in neurology and psychiatry. Parallel validation has been completed to demonstrate the feasibility of repeated home-based use over months to 1 year in AD, frontotemporal dementia, and amyotrophic lateral sclerosis. Some of the tasks are now deployed in phase 1 and phase 2 clinical trials, where monitoring transient changes in cognition is critical. If these tasks can be deployed remotely in patient samples over extended periods (and parallel longitudinal studies [26,27] suggest they can), they could also be used to monitor the efficacy of cognitive symptom improvements in later-phase trials of psychiatric and neurodegenerative therapies.

It has been suggested that using repeatable digital tools to track within-participant delta over time could provide a more reliable and cost-effective approach than relying on noisy group means in CNS clinical trials [6]. Furthermore, regular, low-impact longitudinal measurement in the home setting could represent a trial model less susceptible to the placebo-like effects of the traditional clinic-based model [13] (although this remains to be demonstrated in practice), potentially leading to an improved rate of approval for CNS disease treatments. Our findings, both here and in parallel studies, suggest that the digital test battery described may offer a practical solution to address these key unmet needs.

## Appendix

Multimedia Appendix 1 Wilcoxon signed rank tests for improvement between practice sessions (from session 1 to 2, and 2 to 3, where applicable). Raw and corrected (Holm-Bonferroni) *P* values are shown. Statistically significant practice effects (after correction) are presented in bold.

Multimedia Appendix 2 Linear mixed models for the effect of alcohol across sessions over time: full output table. Raw and corrected (Holm-Bonferroni) *P* values are shown, with 95% CIs. Significant results (after correction) are presented in bold.

## Abbreviations

AD: Alzheimer disease
ADAS-Cog: Alzheimer’s Disease Assessment Scale—Cognitive Subscale
AUDIT: Alcohol Use Disorders Identification Test
BAC: blood alcohol concentration
CANTAB: Cambridge Neuropsychological Test Automated Battery
CDR: Clinical Dementia Rating
CNS: central nervous system
DSST: Digit Symbol Substitution Task
EEG: electroencephalography
MDD: major depressive disorder
MMSE: Mini-Mental Status Examination
MoCA: Montreal Cognitive Assessment
PAL: Paired Associates Learning
PALFAMS: Paired Associates Learning First Attempt Memory Score
PALTEA: Paired Associates Learning Total Errors Adjusted
SDMT: Symbol Digit Modalities Test
VAS: Visual Analog Scale
VPA-I: Verbal Paired Associates—Part 1
WAIS: Wechsler Adult Intelligence Scale
WMS-IV: Wechsler memory scale version 4

## References

[1] Rentz DM, Parra Rodriguez MA, Amariglio R, Stern Y, Sperling R, Ferris S (2013). Promising developments in neuropsychological approaches for the detection of preclinical Alzheimer's disease: a selective review. Alzheimers Res Ther.

[2] Hird N, Ghosh S, Kitano H (2016). Drug Discov Today.

[3] Miller JB, Barr WB (2017). The technology crisis in neuropsychology. Arch Clin Neuropsychol.

[4] Borland E (2024). Advancing the use of brief cognitive tests - establishing norms, clinically relevant changes and predictive models. Doctoral thesis. Lund University.

[5] Lipsmeier F, Simillion C, Bamdadian A, Tortelli R, Byrne LM, Zhang Y, Wolf D, Smith AV, Czech C, Gossens C, Weydt P, Schobel SA, Rodrigues FB, Wild EJ, Lindemann M (2022). A remote digital monitoring platform to assess cognitive and motor symptoms in huntington disease: cross-sectional validation study. J Med Internet Res.

[6] Sliwinski MJ (2008). Measurement‐burst designs for social health research. Social & Personality Psych.

[7] Bora E (2020). Meta-analysis of neurocognitive deficits in unaffected relatives of obsessive-compulsive disorder (OCD): comparison with healthy controls and patients with OCD. Psychol Med.

[8] Gruner P, Pittenger C (2017). Cognitive inflexibility in obsessive-compulsive disorder. Neuroscience.

[9] Perini G, Cotta Ramusino Matteo, Sinforiani E, Bernini S, Petrachi R, Costa A (2019). Cognitive impairment in depression: recent advances and novel treatments. Neuropsychiatr Dis Treat.

[10] Vieta E, Sluth LB, Olsen CK (2018). The effects of vortioxetine on cognitive dysfunction in patients with inadequate response to current antidepressants in major depressive disorder: A short-term, randomized, double-blind, exploratory study versus escitalopram. J Affect Disord.

[11] Colwell MJ, Tagomori H, Chapman S, Gillespie AL, Cowen PJ, Harmer CJ, Murphy SE (2022). Pharmacological targeting of cognitive impairment in depression: recent developments and challenges in human clinical research. Transl Psychiatry.

[12] Baune BT, Sluth LB, Olsen CK (2018). The effects of vortioxetine on cognitive performance in working patients with major depressive disorder: A short-term, randomized, double-blind, exploratory study. J Affect Disord.

[13] Benedetti F, Carlino E, Piedimonte A (2016). Increasing uncertainty in CNS clinical trials: the role of placebo, nocebo, and Hawthorne effects. Lancet Neurol.

[14] Hassenstab J, Aschenbrenner A, Sliwinski M, McDade E, Lim Y, Maruff P (2017). Rapid, remote, and reliable: smartphone-based burst cognitive assessments in Alzheimer’s disease. BrightFocus Foundation.

[15] (2017). Cognitive testing is getting faster and better (coverage of CTAD 2017). AlzForum.

[16] Barbey FM, Farina FR, Buick AR, Danyeli L, Dyer JF, Islam MN, Krylova M, Murphy B, Nolan H, Rueda-Delgado LM, Walter M, Whelan R (2022). Neuroscience from the comfort of your home: repeated, self-administered wireless dry EEG measures brain function with high fidelity. Front Digit Health.

[17] Goldberg TE, Harvey PD, Wesnes KA, Snyder PJ, Schneider LS (2015). Practice effects due to serial cognitive assessment: implications for preclinical Alzheimer's disease randomized controlled trials. Alzheimers Dement (Amst).

[18] Whelan R, Barbey FM, Cominetti MR, Gillan CM, Rosická AM (2022). Developments in scalable strategies for detecting early markers of cognitive decline. Transl Psychiatry.

[19] Curcic J, Vallejo V, Sorinas J, Sverdlov O, Praestgaard J, Piksa M, Deurinck M, Erdemli G, Bügler Maximilian, Tarnanas I, Taptiklis N, Cormack F, Anker R, Massé Fabien, Souillard-Mandar W, Intrator N, Molcho L, Madero E, Bott N, Chambers M, Tamory J, Shulz M, Fernandez G, Simpson W, Robin J, Snædal Jón G, Cha J, Hannesdottir K (2022). Description of the method for evaluating digital endpoints in Alzheimer disease study: protocol for an exploratory, cross-sectional study. JMIR Res Protoc.

[20] (2024). Digital Medicine Society (DiMe) Library of Digital Endpoints. Digital Medicine Society.

[21] Falleti MG, Maruff P, Collie A, Darby DG, McStephen M (2003). Qualitative similarities in cognitive impairment associated with 24 h of sustained wakefulness and a blood alcohol concentration of 0.05%. J Sleep Res.

[22] Falleti MG, Maruff P, Collie A, Darby DG (2006). Practice effects associated with the repeated assessment of cognitive function using the CogState battery at 10-minute, one week and one month test-retest intervals. J Clin Exp Neuropsychol.

[23] Meier I, Buegler M, Harms R, Seixas A, Çöltekin Arzu, Tarnanas I (2021). Using a Digital Neuro Signature to measure longitudinal individual-level change in Alzheimer's disease: the Altoida large cohort study. NPJ Digit Med.

[24] Honey RAE, Turner DC, Honey GD, Sharar SR, Kumaran D, Pomarol-Clotet E, McKenna P, Sahakian BJ, Robbins TW, Fletcher PC (2003). Subdissociative dose ketamine produces a deficit in manipulation but not maintenance of the contents of working memory. Neuropsychopharmacology.

[25] McWilliams EC, Barbey FM, Dyer JF, Islam MN, McGuinness B, Murphy B, Nolan H, Passmore P, Rueda-Delgado LM, Buick AR (2021). Feasibility of repeated assessment of cognitive function in older adults using a wireless, mobile, dry-EEG headset and tablet-based games. Front Psychiatry.

[26] Rueda‐Delgado LM, Buick AR, Hardiman O, Muhammed K, Nolan H, Rowe JB, Waninger S, Murphy B (2023). Feasibility of real world end‐points of functional neurophysiology in Alzheimer’s disease dementia. Alzheimer's & Dementia.

[27] Barbey F, Buick A, Costello E, Dyer JF, Hardiman O, Pender N, Rueda-Delgado L, Murphy B (2023). Patient and technical feasibility of real-world sampling of cognition and functional neurophysiology in ALS and FTD. ResearchGate.

[28] Chamberlain SR, Blackwell AD, Nathan PJ, Hammond G, Robbins TW, Hodges JR, Michael A, Semple JM, Bullmore ET, Sahakian BJ (2011). Differential cognitive deterioration in dementia: a two year longitudinal study. J Alzheimers Dis.

[29] White AM, Matthews DB, Best PJ (2000). Ethanol, memory, and hippocampal function: a review of recent findings. Hippocampus.

[30] Peterson JB, Rothfleisch J, Zelazo PD, Pihl RO (1990). Acute alcohol intoxication and cognitive functioning. J Stud Alcohol.

[31] Yuille JC, Tollestrup PA (1990). Some effects of alcohol on eyewitness memory. J Appl Psychol.

[32] Haut JS, Beckwith BE, Petros TV, Russell S (1989). Gender differences in retrieval from long-term memory following acute intoxication with ethanol. Physiol Behav.

[33] Parker ES, Birnbaum IM, Noble EP (1976). Alcohol and memory: storage and state dependency. Journal of Verbal Learning and Verbal Behavior.

[34] Söderlund H, Parker ES, Schwartz BL, Tulving E (2005). Memory encoding and retrieval on the ascending and descending limbs of the blood alcohol concentration curve. Psychopharmacology (Berl).

[35] Wesnes KA, Garratt C, Wickens M, Gudgeon A, Oliver S (2000). Effects of sibutramine alone and with alcohol on cognitive function in healthy volunteers. Br J Clin Pharmacol.

[36] Cromer JR, Cromer JA, Maruff P, Snyder PJ (2010). Perception of alcohol intoxication shows acute tolerance while executive functions remain impaired. Exp Clin Psychopharmacol.

[37] Casbon TS, Curtin JJ, Lang AR, Patrick CJ (2003). Deleterious effects of alcohol intoxication: diminished cognitive control and its behavioral consequences. J Abnorm Psychol.

[38] Maylor EA, Rabbitt PM (1993). Alcohol, reaction time and memory: a meta-analysis. Br J Psychol.

[39] Faul F, Erdfelder E, Lang A, Buchner A (2007). G*Power 3: a flexible statistical power analysis program for the social, behavioral, and biomedical sciences. Behav Res Methods.

[40] Saunders J B, Aasland O G, Babor T F, de la Fuente J R, Grant M (1993). Development of the Alcohol Use Disorders Identification Test (AUDIT): WHO Collaborative Project on Early Detection of Persons with Harmful Alcohol Consumption--II. Addiction.

[41] Conigrave Km, Hall Wd, Saunders Jb (1995). The AUDIT questionnaire: choosing a cut-off score. Addiction.

[42] Heather N, Partington S, Partington E, Longstaff F, Allsop S, Jankowski M, Wareham H, St Clair Gibson A (2011). Alcohol use disorders and hazardous drinking among undergraduates at English universities. Alcohol Alcohol.

[43] Verhoog S, Dopmeijer JM, de Jonge JM, van der Heijde CM, Vonk P, Bovens RHLM, de Boer MR, Hoekstra T, Kunst AE, Wiers RW, Kuipers MAG (2020). The Use of the Alcohol Use Disorders Identification Test - consumption as an indicator of hazardous alcohol use among university students. Eur Addict Res.

[44] Jaeger J (2018). Digit Symbol Substitution Test: the case for sensitivity over specificity in neuropsychological testing. J Clin Psychopharmacol.

[45] Wechsler D (2008). Wechsler Adult Intelligence Scale - Fourth Edition Administration and Scoring Manual.

[46] Beres CA, Baron A (1981). Improved digit symbol substitution by older women as a result of extended practice. J Gerontol.

[47] Barnett J, Blackwell A, Sahakian B, Robbins T (2016). The Paired Associates Learning (PAL) Test: 30 years of CANTAB translational neuroscience from laboratory to bedside in dementia research. Curr Top Behav Neurosci.

[48] Cormack FK, Taptiklis N, Abbott RA, Anatürk M, Cartland I, Coppieters L, Housden C, Barnett JH (2016). O3‐03‐03: Changes to validity of Online Cognitive Assessment in young and older adults: a comparison to supervised testing using the Cantab battery. Alzheimer's & Dementia.

[49] Fowler K, Saling M, Conway E, Semple J, Louis W (1995). Computerized delayed matching to sample and paired associate performance in the early detection of dementia. Appl Neuropsychol.

[50] Wechsler D (2009). WMS-IV: Wechsler Memory Scale (Fourth Edition).

[51] de Rover M, Pironti VA, McCabe JA, Acosta-Cabronero J, Arana FS, Morein-Zamir S, Hodges JR, Robbins TW, Fletcher PC, Nestor PJ, Sahakian BJ (2011). Hippocampal dysfunction in patients with mild cognitive impairment: a functional neuroimaging study of a visuospatial paired associates learning task. Neuropsychologia.

[52] Campbell S, Macqueen G (2004). The role of the hippocampus in the pathophysiology of major depression. J Psychiatry Neurosci.

[53] Dillon DG, Pizzagalli DA (2018). Mechanisms of memory disruption in depression. Trends Neurosci.

[54] Verhaeghen P, Basak C (2005). Ageing and switching of the focus of attention in working memory: results from a modified N-back task. Q J Exp Psychol A.

[55] Marshall AC, Cooper NR, Segrave R, Geeraert N (2015). The effects of long-term stress exposure on aging cognition: a behavioral and EEG investigation. Neurobiol Aging.

[56] Lett TA, Voineskos AN, Kennedy JL, Levine B, Daskalakis ZJ (2014). Treating working memory deficits in schizophrenia: a review of the neurobiology. Biol Psychiatry.

[57] Nikolin S, Tan YY, Schwaab A, Moffa A, Loo CK, Martin D (2021). An investigation of working memory deficits in depression using the n-back task: a systematic review and meta-analysis. J Affect Disord.

[58] Lawlor-Savage Linette, Goghari Vina M (2016). Dual N-Back working memory training in healthy adults: a randomized comparison to processing speed training. PLoS One.

[59] Der G, Deary IJ (2006). Age and sex differences in reaction time in adulthood: results from the United Kingdom Health and Lifestyle Survey. Psychol Aging.

[60] Draeger.

[61] Sorbello J, Devilly G, Allen C, Hughes L, Brown K (2018). Fuel-cell breathalyser use for field research on alcohol intoxication: an independent psychometric evaluation. PeerJ.

[62] Calculator.net.

[63] Jutten RJ, Harrison J, Lee Meeuw Kjoe PR, Opmeer EM, Schoonenboom NS, de Jong FJ, Ritchie CW, Scheltens P, Sikkes SA (2018). A novel cognitive-functional composite measure to detect changes in early Alzheimer's disease: test-retest reliability and feasibility. Alzheimers Dement (Amst).

[64] Toda T, Parylak SL, Linker SB, Gage FH (2019). The role of adult hippocampal neurogenesis in brain health and disease. Mol Psychiatry.

[65] Clark IA, Kim M, Maguire EA (2018). Verbal paired associates and the hippocampus: the role of scenes. J Cogn Neurosci.

[66] Gignac GE, Szodorai ET (2016). Effect size guidelines for individual differences researchers. Personality and Individual Differences.

[67] Bartels C, Wegrzyn M, Wiedl A, Ackermann V, Ehrenreich H (2010). Practice effects in healthy adults: a longitudinal study on frequent repetitive cognitive testing. BMC Neurosci.

[68] Öhman Fredrik, Hassenstab J, Berron D, Schöll Michael, Papp KV (2021). Current advances in digital cognitive assessment for preclinical Alzheimer's disease. Alzheimers Dement (Amst).

