# Supplementary material for: Temporal Dynamics of Subtle Cognitive Change: Validation of a User-Friendly Multidomain Digital Assessment Using an Alcohol Challenge
Source: J Med Internet Res. 2025 Jun 12;27:e55469. doi: 10.2196/55469 (PMC12203029; doi:10.2196/55469)
Supplement: Multimedia Appendix 1 [file jmir_v27i1e55469_app1.docx]

Appendix 1: Wilcoxon signed-rank tests for improvement between practice sessions (from session 1 to 2, and 2 to 3, where applicable). Raw and corrected (Holm-Bonferroni) *P* values are shown. Statistically significant practice effects (after correction) are presented in bold.

| Task metric | *Z* (2vs1) | *P* (2vs1) | Z (3vs2) | *P* (3vs2) | *P* (2vs1; corrected) | *P* (3vs2; corrected) |
| --- | --- | --- | --- | --- | --- | --- |
| Symbol Swap Total Correct | **51** | **0.005** | **75** | **0.419** | **0.045** | **1** |
| Memory Match Total Correct | 143 | 0.855 | 93 | 0.452 | 1 | 1 |
| Rapid Response Mean Reaction Time (ms) | 120 | 0.263 | 110 | 0.865 | 1 | 1 |
| Rapid Response SD Reaction Time (ms) | 123 | 0.3 | 97 | 0.539 | 1 | 1 |
| Double Take (1-back) Accuracy in Match Trials | 39 | 0.376 | 5.5 | 0.011 | 1 | 0.099 |
| Double Take (2-back) Accuracy (%) | 49.5 | 0.021 | 85 | 0.983 | 0.168 | 1 |
| Double Take (2-back) Accuracy in Match Trials | 46 | 0.025 | 51.5 | 0.623 | 0.175 | 1 |
| Double Take (2-back) Accuracy in Non-match Trials | 34 | 0.076 | 53.5 | 0.709 | 0.456 | 1 |
| Paper DSST Total Correct | 91.5 | 0.155 |  |  | 0.775 |  |
