# Supplementary material for: Temporal Dynamics of Subtle Cognitive Change: Validation of a User-Friendly Multidomain Digital Assessment Using an Alcohol Challenge
Source: J Med Internet Res. 2025 Jun 12;27:e55469. doi: 10.2196/55469 (PMC12203029; doi:10.2196/55469)
Supplement: Multimedia Appendix 2 [file jmir_v27i1e55469_app2.docx]

Appendix 2: Linear mixed models for the effect of alcohol across sessions, over time: full output table. Raw and corrected (Holm-Bonferroni) *P* values are shown, with 95% confidence intervals. Significant results (after correction) are presented in bold.

| Task metric | Effect | *β* coef. | Std.Err. | *t* | *P* | *P* corr. | [0.025 | 0.975] |
| --- | --- | --- | --- | --- | --- | --- | --- | --- |
| Symbol Swap Total Success | Intercept | 39.624 | 1.509 | 26.250 | 0.000 | 0.000 | 36.658 | 42.590 |
|  | C(Gender)[T.M] | -2.439 | 2.308 | -1.057 | 0.291 | 1.000 | -6.975 | 2.097 |
|  | **C(VisitNo)[T.2]** | **2.912** | **0.355** | **8.208** | **0.000** | **0.000** | **2.214** | **3.609** |
|  | C(SessionType)[T.B_alc] | 0.067 | 1.002 | 0.067 | 0.947 | 1.000 | -1.903 | 2.036 |
|  | C(Timeline)[T.+045 mins] | 1.133 | 1.002 | 1.131 | 0.259 | 1.000 | -0.836 | 3.103 |
|  | C(Timeline)[T.+090 mins] | 0.667 | 1.002 | 0.665 | 0.506 | 1.000 | -1.303 | 2.636 |
|  | C(Timeline)[T.+120 mins] | 1.167 | 1.002 | 1.164 | 0.245 | 1.000 | -0.803 | 3.136 |
|  | C(Timeline)[T.+180 mins] | 2.267 | 1.002 | 2.262 | 0.024 | 0.290 | 0.297 | 4.236 |
|  | C(Timeline)[T.+240 mins] | 1.167 | 1.002 | 1.164 | 0.245 | 1.000 | -0.803 | 3.136 |
|  | C(Timeline)[T.+300 mins] | 2.067 | 1.002 | 2.062 | 0.040 | 0.397 | 0.097 | 4.036 |
|  | C(Timeline)[T.Initial dose] | 0.267 | 1.002 | 0.266 | 0.790 | 1.000 | -1.703 | 2.236 |
|  | **C(SessionType)[T.B_alc]:C(Timeline)[T.+045 mins]** | **-6.100** | **1.417** | **-4.304** | **0.000** | **0.000** | **-8.885** | **-3.315** |
|  | **C(SessionType)[T.B_alc]:C(Timeline)[T.+090 mins]** | **-5.367** | **1.417** | **-3.787** | **0.000** | **0.003** | **-8.152** | **-2.582** |
|  | C(SessionType)[T.B_alc]:C(Timeline)[T.+120 mins] | -3.067 | 1.417 | -2.164 | 0.031 | 0.341 | -5.852 | -0.282 |
|  | **C(SessionType)[T.B_alc]:C(Timeline)[T.+180 mins]** | **-4.167** | **1.417** | **-2.940** | **0.003** | **0.048** | **-6.952** | **-1.382** |
|  | C(SessionType)[T.B_alc]:C(Timeline)[T.+240 mins] | -0.541 | 1.424 | -0.380 | 0.704 | 1.000 | -3.339 | 2.257 |
|  | C(SessionType)[T.B_alc]:C(Timeline)[T.+300 mins] | -1.700 | 1.417 | -1.200 | 0.231 | 1.000 | -4.485 | 1.085 |
|  | C(SessionType)[T.B_alc]:C(Timeline)[T.Initial dose] | -3.433 | 1.417 | -2.423 | 0.016 | 0.205 | -6.218 | -0.648 |
|  | UserID Var | 34.580 | 2.524 | N/A | N/A | N/A | N/A | N/A |
| Memory Match Total Correct | Intercept | 31.222 | 4.217 | 7.403 | 0.000 | 0.000 | 22.898 | 39.547 |
|  | C(Sex)[T.M] | 2.733 | 5.633 | 0.485 | 0.628 | 1.000 | -8.385 | 13.852 |
|  | C(VisitNo)[T.2] | 2.467 | 2.192 | 1.125 | 0.262 | 1.000 | -1.861 | 6.794 |
|  | C(SessionType)[T.B_alc] | 0.400 | 3.797 | 0.105 | 0.916 | 1.000 | -7.095 | 7.895 |
|  | C(Timeline)[T.+045 mins] | -3.467 | 3.797 | -0.913 | 0.363 | 1.000 | -10.962 | 4.029 |
|  | C(Timeline)[T.+300 mins] | -3.833 | 3.797 | -1.009 | 0.314 | 1.000 | -11.329 | 3.662 |
|  | **C(SessionType)[T.B_alc]:C(Timeline)[T.+045 mins]** | **-18.400** | **5.370** | **-3.426** | **0.001** | **0.005** | **-29.000** | **-7.800** |
|  | C(SessionType)[T.B_alc]:C(Timeline)[T.+300 mins] | -5.533 | 5.370 | -1.030 | 0.304 | 1.000 | -16.133 | 5.067 |
|  | UserID Var | 175.473 | 4.201 |  |  |  |  |  |
| Rapid Response Mean Reaction Time (ms) | Intercept | 26.470 | 0.190 | 139.064 | 0.000 | 0.000 | 26.096 | 26.844 |
|  | C(Sex)[T.M] | -0.012 | 0.284 | -0.042 | 0.966 | 1.000 | -0.571 | 0.547 |
|  | C(VisitNo)[T.2] | 0.124 | 0.048 | 2.581 | 0.010 | 0.142 | 0.030 | 0.219 |
|  | C(SessionType)[T.B_alc] | 0.008 | 0.136 | 0.057 | 0.954 | 1.000 | -0.260 | 0.276 |
|  | C(Timeline)[T.Initial dose] | 0.065 | 0.136 | 0.476 | 0.635 | 1.000 | -0.203 | 0.333 |
|  | C(Timeline)[T.+045 mins] | 0.037 | 0.136 | 0.271 | 0.786 | 1.000 | -0.231 | 0.305 |
|  | C(Timeline)[T.+090 mins] | -0.026 | 0.136 | -0.189 | 0.850 | 1.000 | -0.294 | 0.242 |
|  | C(Timeline)[T.+120 mins] | -0.023 | 0.136 | -0.165 | 0.869 | 1.000 | -0.291 | 0.246 |
|  | C(Timeline)[T.+180 mins] | -0.043 | 0.136 | -0.315 | 0.753 | 1.000 | -0.311 | 0.225 |
|  | C(Timeline)[T.+240 mins] | 0.063 | 0.136 | 0.465 | 0.642 | 1.000 | -0.205 | 0.331 |
|  | C(Timeline)[T.+300 mins] | 0.214 | 0.136 | 1.566 | 0.118 | 1.000 | -0.054 | 0.482 |
|  | C(SessionType)[T.B_alc]:C(Timeline)[T.Initial dose] | 0.267 | 0.193 | 1.385 | 0.167 | 1.000 | -0.112 | 0.646 |
|  | **C(SessionType)[T.B_alc]:C(Timeline)[T.+045 mins]** | **0.783** | **0.193** | **4.060** | **0.000** | **0.001** | **0.404** | **1.162** |
|  | **C(SessionType)[T.B_alc]:C(Timeline)[T.+090 mins]** | **0.816** | **0.193** | **4.232** | **0.000** | **0.000** | **0.437** | **1.196** |
|  | **C(SessionType)[T.B_alc]:C(Timeline)[T.+120 mins]** | **0.607** | **0.193** | **3.145** | **0.002** | **0.027** | **0.228** | **0.986** |
|  | C(SessionType)[T.B_alc]:C(Timeline)[T.+180 mins] | 0.471 | 0.193 | 2.443 | 0.015 | 0.194 | 0.092 | 0.850 |
|  | C(SessionType)[T.B_alc]:C(Timeline)[T.+240 mins] | 0.231 | 0.193 | 1.199 | 0.231 | 1.000 | -0.148 | 0.610 |
|  | C(SessionType)[T.B_alc]:C(Timeline)[T.+300 mins] | 0.117 | 0.193 | 0.606 | 0.545 | 1.000 | -0.262 | 0.496 |
|  | UserID Var | 0.521 | 0.281 |  |  |  |  |  |
| Rapid Response SD Reaction Time (ms) | Intercept | 19.265 | 0.584 | 32.978 | 0.000 | 0.000 | 18.117 | 20.413 |
|  | C(Sex)[T.M] | 0.336 | 0.719 | 0.468 | 0.640 | 1.000 | -1.076 | 1.748 |
|  | C(VisitNo)[T.2] | 0.229 | 0.206 | 1.112 | 0.267 | 1.000 | -0.176 | 0.633 |
|  | C(SessionType)[T.B_alc] | -0.809 | 0.582 | -1.390 | 0.165 | 1.000 | -1.952 | 0.334 |
|  | C(Timeline)[T.Initial dose] | -0.569 | 0.582 | -0.979 | 0.328 | 1.000 | -1.712 | 0.574 |
|  | C(Timeline)[T.+045 mins] | -0.498 | 0.582 | -0.856 | 0.393 | 1.000 | -1.641 | 0.645 |
|  | C(Timeline)[T.+090 mins] | -0.696 | 0.582 | -1.197 | 0.232 | 1.000 | -1.839 | 0.447 |
|  | C(Timeline)[T.+120 mins] | -1.122 | 0.582 | -1.930 | 0.054 | 0.597 | -2.265 | 0.021 |
|  | C(Timeline)[T.+180 mins] | -1.221 | 0.582 | -2.099 | 0.036 | 0.437 | -2.364 | -0.078 |
|  | C(Timeline)[T.+240 mins] | -0.731 | 0.582 | -1.257 | 0.209 | 1.000 | -1.874 | 0.412 |
|  | C(Timeline)[T.+300 mins] | 0.135 | 0.582 | 0.232 | 0.817 | 1.000 | -1.008 | 1.278 |
|  | C(SessionType)[T.B_alc]:C(Timeline)[T.Initial dose] | 1.383 | 0.823 | 1.681 | 0.093 | 0.934 | -0.234 | 2.999 |
|  | C(SessionType)[T.B_alc]:C(Timeline)[T.+045 mins] | 2.207 | 0.823 | 2.683 | 0.008 | 0.106 | 0.590 | 3.823 |
|  | **C(SessionType)[T.B_alc]:C(Timeline)[T.+090 mins]** | **3.971** | **0.823** | **4.828** | **0.000** | **0.000** | **2.355** | **5.587** |
|  | **C(SessionType)[T.B_alc]:C(Timeline)[T.+120 mins]** | **2.735** | **0.823** | **3.325** | **0.001** | **0.014** | **1.119** | **4.351** |
|  | **C(SessionType)[T.B_alc]:C(Timeline)[T.+180 mins]** | **2.767** | **0.823** | **3.364** | **0.001** | **0.013** | **1.151** | **4.383** |
|  | C(SessionType)[T.B_alc]:C(Timeline)[T.+240 mins] | 2.171 | 0.823 | 2.640 | 0.009 | 0.112 | 0.555 | 3.788 |
|  | C(SessionType)[T.B_alc]:C(Timeline)[T.+300 mins] | 1.089 | 0.823 | 1.323 | 0.186 | 1.000 | -0.528 | 2.705 |
|  | UserID Var | 3.125 | 0.421 |  |  |  |  |  |
| Double Take (1-back) Accuracy in Match Trials | Intercept | 19.974 | 0.070 | 284.707 | 0.000 | 0.000 | 19.836 | 20.113 |
|  | C(Sex)[T.M] | -0.004 | 0.062 | -0.062 | 0.951 | 1.000 | -0.127 | 0.119 |
|  | C(VisitNo)[T.2] | -0.102 | 0.049 | -2.074 | 0.040 | 0.237 | -0.199 | -0.005 |
|  | C(SessionType)[T.B_alc] | -0.061 | 0.085 | -0.717 | 0.474 | 1.000 | -0.229 | 0.107 |
|  | C(Timeline)[T.+045 mins] | 0.002 | 0.085 | 0.021 | 0.983 | 1.000 | -0.166 | 0.170 |
|  | C(Timeline)[T.+300 mins] | -0.127 | 0.085 | -1.497 | 0.136 | 0.681 | -0.295 | 0.041 |
|  | **C(SessionType)[T.B_alc]:C(Timeline)[T.+045 mins]** | **-0.376** | **0.120** | **-3.127** | **0.002** | **0.015** | **-0.614** | **-0.139** |
|  | C(SessionType)[T.B_alc]:C(Timeline)[T.+300 mins] | -0.025 | 0.120 | -0.204 | 0.838 | 1.000 | -0.262 | 0.213 |
|  | UserID Var | 0.008 | 0.023 |  |  |  |  |  |
| Double Take (2-back) Accuracy (%) | Intercept | 19.426 | 0.110 | 176.781 | 0.000 | 0.000 | 19.209 | 19.643 |
|  | C(Sex)[T.M] | 0.195 | 0.153 | 1.278 | 0.203 | 1.000 | -0.106 | 0.497 |
|  | C(VisitNo)[T.2] | 0.049 | 0.053 | 0.923 | 0.357 | 1.000 | -0.056 | 0.155 |
|  | C(SessionType)[T.B_alc] | -0.007 | 0.092 | -0.074 | 0.941 | 1.000 | -0.189 | 0.176 |
|  | C(Timeline)[T.+045 mins] | -0.051 | 0.092 | -0.548 | 0.584 | 1.000 | -0.233 | 0.132 |
|  | C(Timeline)[T.+300 mins] | 0.105 | 0.092 | 1.135 | 0.258 | 1.000 | -0.078 | 0.287 |
|  | **C(SessionType)[T.B_alc]:C(Timeline)[T.+045 mins]** | **-0.385** | **0.131** | **-2.947** | **0.004** | **0.026** | **-0.643** | **-0.127** |
|  | C(SessionType)[T.B_alc]:C(Timeline)[T.+300 mins] | -0.148 | 0.131 | -1.129 | 0.260 | 1.000 | -0.406 | 0.110 |
|  | UserID Var | 0.135 | 0.127 |  |  |  |  |  |
| Double Take (2-back) Accuracy in Match Trials | Intercept | 19.057 | 0.275 | 69.221 | 0.000 | 0.000 | 18.514 | 19.601 |
|  | C(Sex)[T.M] | 0.362 | 0.329 | 1.099 | 0.273 | 1.000 | -0.288 | 1.011 |
|  | C(VisitNo)[T.2] | 0.275 | 0.163 | 1.689 | 0.093 | 0.558 | -0.046 | 0.596 |
|  | C(SessionType)[T.B_alc] | -0.230 | 0.282 | -0.816 | 0.415 | 1.000 | -0.786 | 0.326 |
|  | C(Timeline)[T.+045 mins] | -0.259 | 0.282 | -0.919 | 0.359 | 1.000 | -0.815 | 0.297 |
|  | C(Timeline)[T.+300 mins] | 0.000 | 0.282 | 0.000 | 1.000 | 1.000 | -0.556 | 0.556 |
|  | C(SessionType)[T.B_alc]:C(Timeline)[T.+045 mins] | -0.851 | 0.398 | -2.135 | 0.034 | 0.239 | -1.637 | -0.064 |
|  | C(SessionType)[T.B_alc]:C(Timeline)[T.+300 mins] | -0.094 | 0.398 | -0.236 | 0.814 | 1.000 | -0.881 | 0.693 |
|  | UserID Var | 0.523 | 0.193 |  |  |  |  |  |
| Paper DSST Total Correct | Intercept | 87.970 | 3.436 | 25.601 | 0.000 | 0.000 | 81.217 | 94.722 |
|  | C(Sex)[T.M] | -9.653 | 5.497 | -1.756 | 0.080 | 0.798 | -20.456 | 1.150 |
|  | **C(VisitNo)[T.2]** | **5.629** | **0.658** | **8.549** | **0.000** | **0.000** | **4.335** | **6.923** |
|  | C(SessionType)[T.B_alc] | 1.600 | 1.862 | 0.859 | 0.391 | 1.000 | -2.060 | 5.260 |
|  | C(Timeline)[T.Initial dose] | 0.033 | 1.862 | 0.018 | 0.986 | 1.000 | -3.626 | 3.693 |
|  | C(Timeline)[T.+045 mins] | -0.800 | 1.862 | -0.430 | 0.668 | 1.000 | -4.460 | 2.860 |
|  | C(Timeline)[T.+090 mins] | 1.300 | 1.862 | 0.698 | 0.486 | 1.000 | -2.360 | 4.960 |
|  | C(Timeline)[T.+120 mins] | 3.567 | 1.862 | 1.915 | 0.056 | 0.673 | -0.093 | 7.226 |
|  | C(Timeline)[T.+180 mins] | 2.533 | 1.862 | 1.360 | 0.174 | 1.000 | -1.126 | 6.193 |
|  | C(Timeline)[T.+240 mins] | 2.167 | 1.862 | 1.163 | 0.245 | 1.000 | -1.493 | 5.826 |
|  | C(Timeline)[T.+300 mins] | 0.733 | 1.862 | 0.394 | 0.694 | 1.000 | -2.926 | 4.393 |
|  | C(SessionType)[T.B_alc]:C(Timeline)[T.Initial dose] | -5.833 | 2.634 | -2.215 | 0.027 | 0.354 | -11.009 | -0.658 |
|  | **C(SessionType)[T.B_alc]:C(Timeline)[T.+045 mins]** | **-9.733** | **2.634** | **-3.696** | **0.000** | **0.004** | **-14.909** | **-4.558** |
|  | **C(SessionType)[T.B_alc]:C(Timeline)[T.+090 mins]** | **-10.767** | **2.634** | **-4.088** | **0.000** | **0.001** | **-15.942** | **-5.591** |
|  | **C(SessionType)[T.B_alc]:C(Timeline)[T.+120 mins]** | **-8.900** | **2.634** | **-3.379** | **0.001** | **0.011** | **-14.076** | **-3.724** |
|  | C(SessionType)[T.B_alc]:C(Timeline)[T.+180 mins] | -4.933 | 2.634 | -1.873 | 0.062 | 0.679 | -10.109 | 0.242 |
|  | C(SessionType)[T.B_alc]:C(Timeline)[T.+240 mins] | -3.133 | 2.634 | -1.190 | 0.235 | 1.000 | -8.309 | 2.042 |
|  | C(SessionType)[T.B_alc]:C(Timeline)[T.+300 mins] | 3.733 | 2.634 | 1.417 | 0.157 | 1.000 | -1.442 | 8.909 |
|  | UserID Var | 198.212 | 7.702 |  |  |  |  |  |
| PAL First Attempt Memory Score (PALFAMS) | Intercept | 15.344 | 0.829 | 18.504 | 0.000 | 0.000 | 13.708 | 16.981 |
|  | C(Sex)[T.M] | 0.333 | 1.131 | 0.295 | 0.769 | 1.000 | -1.899 | 2.565 |
|  | C(VisitNo)[T.2] | 0.089 | 0.417 | 0.213 | 0.832 | 1.000 | -0.735 | 0.913 |
|  | C(SessionType)[T.B_alc] | 0.600 | 0.723 | 0.830 | 0.408 | 1.000 | -0.827 | 2.027 |
|  | C(Timeline)[T.+045 mins] | 0.900 | 0.723 | 1.245 | 0.215 | 0.860 | -0.527 | 2.327 |
|  | C(Timeline)[T.+300 mins] | 1.500 | 0.723 | 2.075 | 0.040 | 0.198 | 0.073 | 2.927 |
|  | **C(SessionType)[T.B_alc]:C(Timeline)[T.+045 mins]** | **-5.200** | **1.022** | **-5.086** | **0.000** | **0.000** | **-7.218** | **-3.182** |
|  | C(SessionType)[T.B_alc]:C(Timeline)[T.+300 mins] | -2.333 | 1.022 | -2.282 | 0.024 | 0.142 | -4.352 | -0.315 |
|  | UserID Var | 7.219 | 0.889 |  |  |  |  |  |
| PAL Total Errors Adjusted (PALTEA28) | Intercept | 7.239 | 2.482 | 2.917 | 0.004 | 0.028 | 2.340 | 12.138 |
|  | C(Sex)[T.M] | -2.300 | 3.640 | -0.632 | 0.528 | 1.000 | -9.484 | 4.884 |
|  | C(VisitNo)[T.2] | 0.522 | 1.078 | 0.484 | 0.629 | 1.000 | -1.606 | 2.650 |
|  | C(SessionType)[T.B_alc] | -1.133 | 1.867 | -0.607 | 0.545 | 1.000 | -4.819 | 2.553 |
|  | C(Timeline)[T.+045 mins] | -1.833 | 1.867 | -0.982 | 0.328 | 1.000 | -5.519 | 1.853 |
|  | C(Timeline)[T.+300 mins] | -1.500 | 1.867 | -0.803 | 0.423 | 1.000 | -5.186 | 2.186 |
|  | **C(SessionType)[T.B_alc]:C(Timeline)[T.+045 mins]** | **15.100** | **2.641** | **5.718** | **0.000** | **0.000** | **9.887** | **20.313** |
|  | C(SessionType)[T.B_alc]:C(Timeline)[T.+300 mins] | 3.867 | 2.641 | 1.464 | 0.145 | 0.870 | -1.346 | 9.079 |
|  | UserID Var | 79.597 | 3.567 |  |  |  |  |  |
